# Sound effects: paradigm gene therapy approach installs hearing in children and adolescents born deaf

**DOI:** 10.1038/s41392-026-02879-y

**Published:** 2026-07-23

**Authors:** Juliane W. Schott, Michael Morgan, Axel Schambach

**Affiliations:** 1 Institute of Experimental Hematology, Hannover Medical School, Hannover, Germany; 2 REBIRTH Research Center for Translational Regenerative Medicine, Hannover Medical School, Hannover, Germany; 3 Division of Hematology / Oncology, Boston Children’s Hospital, Harvard Medical School, Boston, MA USA

**Keywords:** Molecular medicine, Genetics

The clinical study CHORD, recently published in The New England Journal of Medicine by Valayannopoulos et al.^1^ demonstrated proof-of-principle for children with profound deafness to hear for the first time, enabled by intracochlear infusion of the dual adeno-associated virus 1 (AAV1) gene therapy DB-OTO. This is the first study determining efficacy as the primary endpoint of a gene therapy trial on hearing loss and represents an important addition to the growing clinical evidence that forms of genetic deafness can be effectively and safely treated with gene therapy. Based on the findings of the trial, DB-OTO was granted FDA approval in April 2026 under the name Otarmeni as the first-ever gene therapy for the treatment of congenital hearing loss.

Normal hearing starts between 0 and 25 decibels (dB). Worldwide, 430 million people are affected by disabling hearing loss of >35 dB. The current study treated patients with profound hearing loss (>90 dB) for whom cochlear implants (CI) are the only current treatment option. However, CI only address the symptoms, but not the cause of the hearing loss, bypassing the action of cochlear hair cells (HC) by directly contacting spiral ganglion neurons (SGN) with an electrode array, and thus neither restore nor reach the quality of natural hearing.

Over 60% of sensorineural hearing loss cases are caused by mutations found in >150 different genes, including otoferlin (*OTOF*). All 12 children (9 females, 3 males, 0.9–16.4 years of age) in this study had biallelic *OTOF* pathogenic variants underlying autosomal recessive deafness 9 (DFNB9), a common form of auditory neuropathy.

**Fig. 1:**
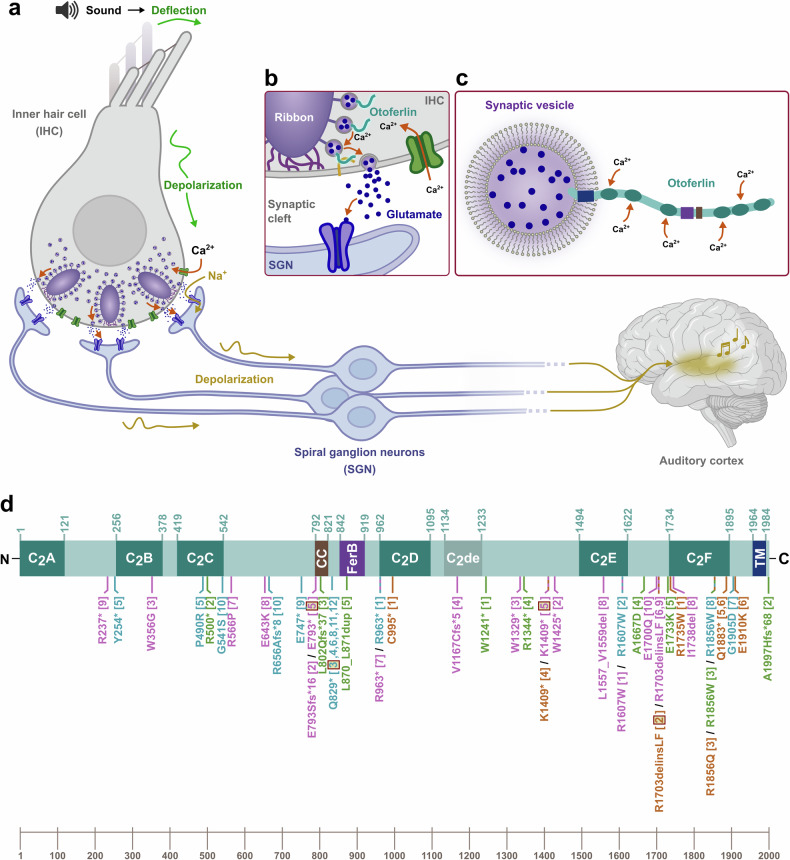
Overview of the role of otoferlin in the hearing cascade, with the position and type of mutations across all DFNB9 patients treated with OTOF gene therapy shown. **a** Inner hair cells (IHC) are the central sensors of the cochlea that transduce the mechanical stimulus of sound into an electrical signal, which they transmit to spiral ganglion neurons (SGN) via a special type of synapse known as a ribbon-synapse. Upon encounter of sound, a traveling wave is caused within the fluid that fills the cochlea, leading to a deflection of the stereocilia on top of the hair cells and subsequent opening of mechanically gated ion channels. K^+^ ion influx causes depolarization of the cells, followed by the opening of Ca^2+^ channels, which trigger the release of the neurotransmitter glutamate into the synaptic cleft through fusion of synaptic vesicles at active zones. As a structural hallmark of IHC, the vesicles are tethered to an electron-dense body, the ribbon, formed by several proteins and anchored to the presynaptic membrane. The ribbon tethers a halo of synaptic vesicles close to the active zones so that the pool of primed and docked vesicles can continuously be sustained, and neurotransmitter can be released in a rapid, temporally precise, and sustained manner. This allows IHC to respond to a large dynamic range of sound intensity encoded by graded receptor potentials and translated into a rapid and precise discharge rate at the synapse, encoding information on intensity and timing of the initial sound stimulus. The released glutamate causes the opening of Na^+^ channels at the postsynaptic terminals of SGN, which depolarize and project the signal to the auditory cortex via the auditory nerve. **b** Otoferlin, encoded by the *OTOF* gene, acts as the major Ca^2+^ sensor of IHC that regulates vesicle exocytosis. It has essential functions at several stages of the vesicle cycle, with implicated roles in docking, priming, fusion, endocytosis, and replenishment of vesicles at active zones. Upon Ca^2+^ binding, otoferlin is thought to interact with SNARE complex proteins (yellow). **c** Otoferlin is anchored via its C-terminal transmembrane domain to synaptic vesicles and/or the plasma membrane, and contains several domains which target membrane surfaces following Ca^2+^ binding. **d** Overview of the 1997 amino acid otoferlin protein, which contains six C_2_ domains (C_2_A–F), out of which all except for C_2_A are implicated in Ca^2+^ and phospholipid binding, a coiled coil (CC) domain, discussed to operate similarly to SNARE motifs, a FerB domain (containing a motif shared by proteins of the ferlin family), and a single C-terminal transmembrane (TM) domain. A seventh C_2_ domain (C_2_de) is predicted. The amino acid positions of the domains within the otoferlin protein are indicated in teal above the image. The position and type of mutation within the otoferlin protein for the participants of the different OTOF gene therapy trials is displayed color-coded for the different trials in turquoise^1^, pink^2^, green^3^, and orange^4^. From the most recent trial,^5^ no detailed information on the patients’ genotypes was available. The trial-internal participant ID (number) is indicated in brackets for each mutation. *Nonsense mutation. Alleles with intronic mutations are not displayed. Participants who did not show any improvement upon gene therapy are highlighted with a red-framed yellow box. Remarkably, DB-OTO and previous OTOF gene therapy products improved hearing in participants with different positions and types of OTOF mutations, which were located to different domains of the OTOF protein and included both nonsense, missense, frameshift, and intronic mutations, resulting either in a truncated protein or a protein with one to several amino acid exchanges, insertions, or deletions. This suggests a general applicability of the treatment independent of the highly diverse genetic contexts found in the *OTOF* locus across DFNB9 patients (>200 pathogenic variants identified). The figure was created using Inkscape. The illustration of the brain was created with BioRender.com

Hearing is a complex process in which sound waves are translated into electrochemical signals interpreted by the auditory cortex as sounds (Fig. 1). Pathogenic *OTOF* variants disrupt the critical step of synaptic transmission between cochlear inner HC and SGN, halting the transmission of acoustic information to the brain.

To deliver intact *OTOF*, DB-OTO is composed of two AAV1 vectors that separately encode for the 5’ and 3’ portions of *OTOF*, and that need to co-transduce the target cell and recombine to generate the full 1997 amino acid otoferlin protein. This dual AAV approach was necessary to deliver the large 5994 bp *OTOF* cDNA and all necessary accompanying sequences, as it exceeds the natural AAV cargo capacity. As AAV1 generally transduces multiple cell types within the cochlea, the authors of this study used the HC-specific *Myo15* promoter as a transcriptional targeting approach. This may help limit potential phenotoxicity caused by off-target transduction.

DB-OTO gene therapy installed synaptic transmission, leading to measurable electrical currents projected to the brain upon sound encounter and to hearing sensation by the patients. Positive changes were observed in 11/12 patients, 9/12 patients reached a hearing level that ended the need for a CI (<70 dB), and 3/12 patients achieved normal acoustic hearing sensitivity within 6 months of treatment. DB-OTO confirmed the primary safety and secondary efficacy observations of previous clinical OTOF gene therapy trials,^2–4^ which also used dual AAV vectors (including different serotypes and vector designs). Several common findings emerged across all studies: (i) the benefit of DFNB9 gene therapy is not restricted to patients with early intervention, (ii) repeated administration into the same ear is possible, tolerated and can improve therapeutic outcome in case of insufficient response to the first dose, (iii) bilateral treatment is advocated for best responses, (iv) the clinical effects after a single injection are durable through follow-up end points of 24 or 48 weeks, and (v) the gene therapy is compatible with subsequent CI.

The positive outcomes observed from infants and toddlers to young adults may reflect that the cochlear structure of DFNB9 patients remains intact even into adulthood, which provides a larger window for therapeutic intervention in DFNB9, in contrast to hearing loss due to pathogenic variants in other genes (e.g., *MYO7A*, *GJB2*) that lead to cell degeneration. Reasons for failure of the treatment in a few patients (1 patient in the DB-OTO study) are currently unknown and hypothesized to potentially result from delivery failure during surgery. Larger cohorts are required to elucidate factors determining success or failure and the parameters governing the magnitude of clinical effects. The most recent study applying AAV-OTOF gene therapy included a larger patient number (42 participants), an extended age range (0.8–32.3 years), and a longer follow-up (2.5 years) than previous trials. Preliminary predictors associated with a higher degree of recovery included age at treatment <18 years, a higher number of baseline distortion product otoacoustic emissions (DPOAE), a measure of outer HC integrity and function, and biallelic non-truncated *OTOF* variants. Progressive hearing improvement was observed throughout the monitored 2.5 years after treatment, but these findings need to be confirmed in future trials.^5^

Compared to earlier clinical OTOF trials, DB-OTO was administered at much larger injection volumes (240 µL/ear versus 30–50 µL/ear). Lateral semicircular canal fenestration, in addition to intracochlear infusion to the round window, was done to reduce the risk of trauma upon application of this large volume into the restricted cochlear space. The number of adverse events (AE) in the DB-OTO trial was similar to previous OTOF trials, and these were transient and resolved without sequelae. Thus, even though the injection volume exceeded the estimated maximum safe volume of 71 µL per ear,^2^ the approach appeared safe, with the only two serious AE not related to the gene therapy. Interestingly, the presence of pre-existing serum anti-AAV neutralizing antibodies did not affect treatment efficacy, indicating the broad applicability of the approach to patients with different AAV immune status.

Limitations of the study include the small sample size and the current short follow-up. A 5-year follow-up is planned, but a benchmark for optimum follow-up times for inner ear gene therapies remains to be defined. Seventeen of the AE observed in the DB-OTO trial were related to surgical delivery, so that administration protocols may be optimized for future studies. Due to potential vector escape to sites outside the inner ear, the panel of parameters evaluated during in vivo gene therapy follow-up needs to be carefully considered for safety analysis.

Important future steps for broader and more precise application include the revision of screening regimens. In DFNB9, outer HC are initially functional, and patients often have normal DPOAE, precluding diagnosis in DPOAE-based newborn screenings. Therefore, we should reconsider screening methods to more comprehensively identify children who already suffer from hearing loss or who are at risk of developing hearing loss, such as early genetic screening to identify pathogenic variants known to cause hearing loss.

Beyond OTOF, gene therapy has the potential to be broadened to treat other forms of genetic hearing loss. Here, established vector platforms and administration routes could be employed. However, it will have to be determined if clinical success will be similar to the OTOF trials, and outcomes may depend on whether the pathogenic variant(s) generate dominant negative mutant proteins, whether the target cells degenerate or are developmentally essential for proper formation or function of the cochlea, its structures, or resident cell types, and whether overexpression causes phenotoxicity in non-target cells. Alternative gene therapy platforms may be explored to fine-tune cargo delivery and expression, increase efficiency, especially for the delivery of large coding sequences on a single vector (e.g., lentiviral vectors as shown for *Myo7a* in a pre-clinical deafness mouse model)^6^, or correct mutations on site or correct/suppress mutational effects in the transcribed mRNA using gene editing or RNA editing approaches, respectively.
